# Supplementary material for: Comparisons of Post-Load Glucose at Different Time Points for Identifying High Risks of MASLD Progression
Source: Nutrients. 2024 Dec 31;17(1):152. doi: 10.3390/nu17010152 (PMC11723153; doi:10.3390/nu17010152)
Supplement: Supplementary file 1 [file nutrients-17-00152-s001.zip › nutrients-3351449-supplementary.pdf]

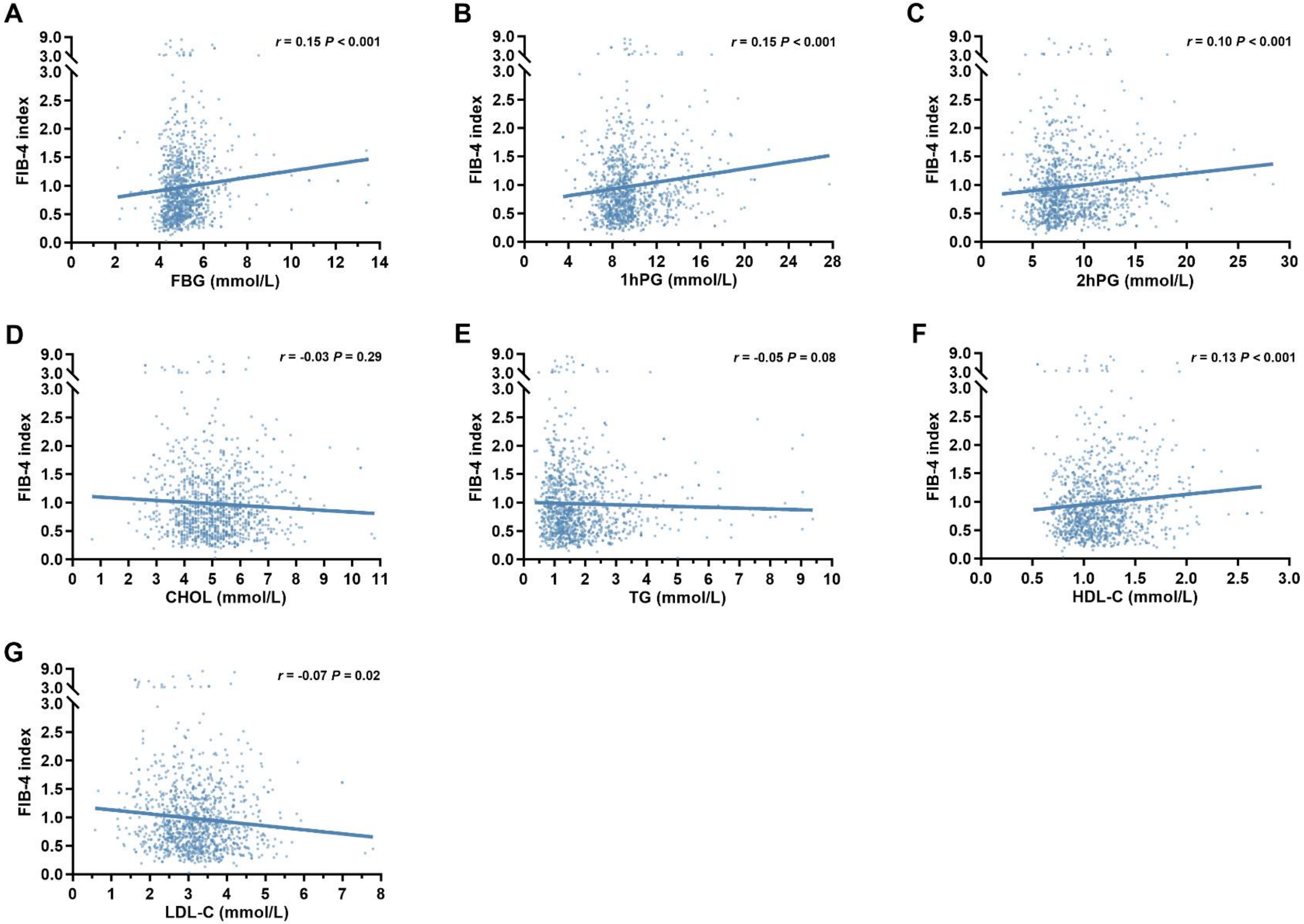

Figure S1. Correlation between clinical parameters, including FBG (A), 1hPG (B), 2hPG (C), CHOL (D), TG (E), HDL-C (F), and LDL-C (G), with the FIB-4 index in MASLD patients. Abbreviation: FBG, fasting blood glucose; 1hPG, 1-hour post load plasma glucose; 2hPG, 2-hour post load plasma glucose; CHOL, cholesterol; TG, triglyceride; HDL-C, high-density lipoprotein cholesterol; LDL-C, low-density lipoprotein cholesterol; FIB-4 index, fibrosis-4 index. The correlation coefficients r and p were obtained by spearman correlation analysis.

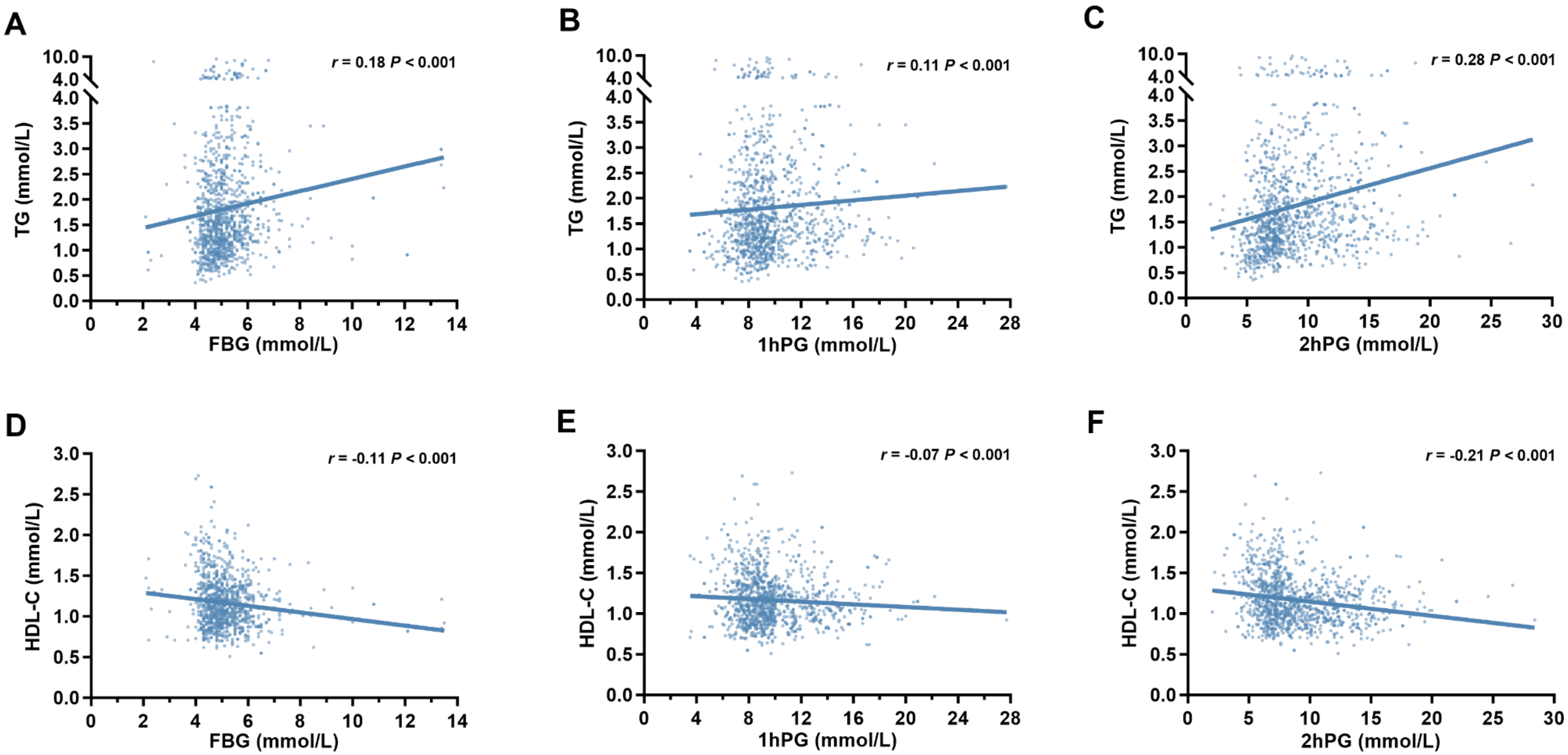

Figure S2. Correlation between glucose levels and triglyceride and high-density lipoprotein cholesterol levels. The correlation coefficients r and p were obtained by spearman correlation analysis. (A) Correlation of FBG level with TG in MASLD patients. (B) Correlation of 1hPG level with TG in MASLD patients. (C) Correlation of 2hPG level with TG in MASLD patients. (D) Correlation of FBG level with HDL-C in MASLD patients. (E) Correlation of 1hPG level with HDL-C in MASLD patients. (F) Correlation of 2hPG level with HDL-C in MASLD patients. Abbreviation: FBG, fasting blood glucose; 1hPG, 1-hour post load plasma glucose; 2hPG, 2-hour post load plasma glucose; TG, triglyceride; HDL-C, high-density lipoprotein cholesterol.

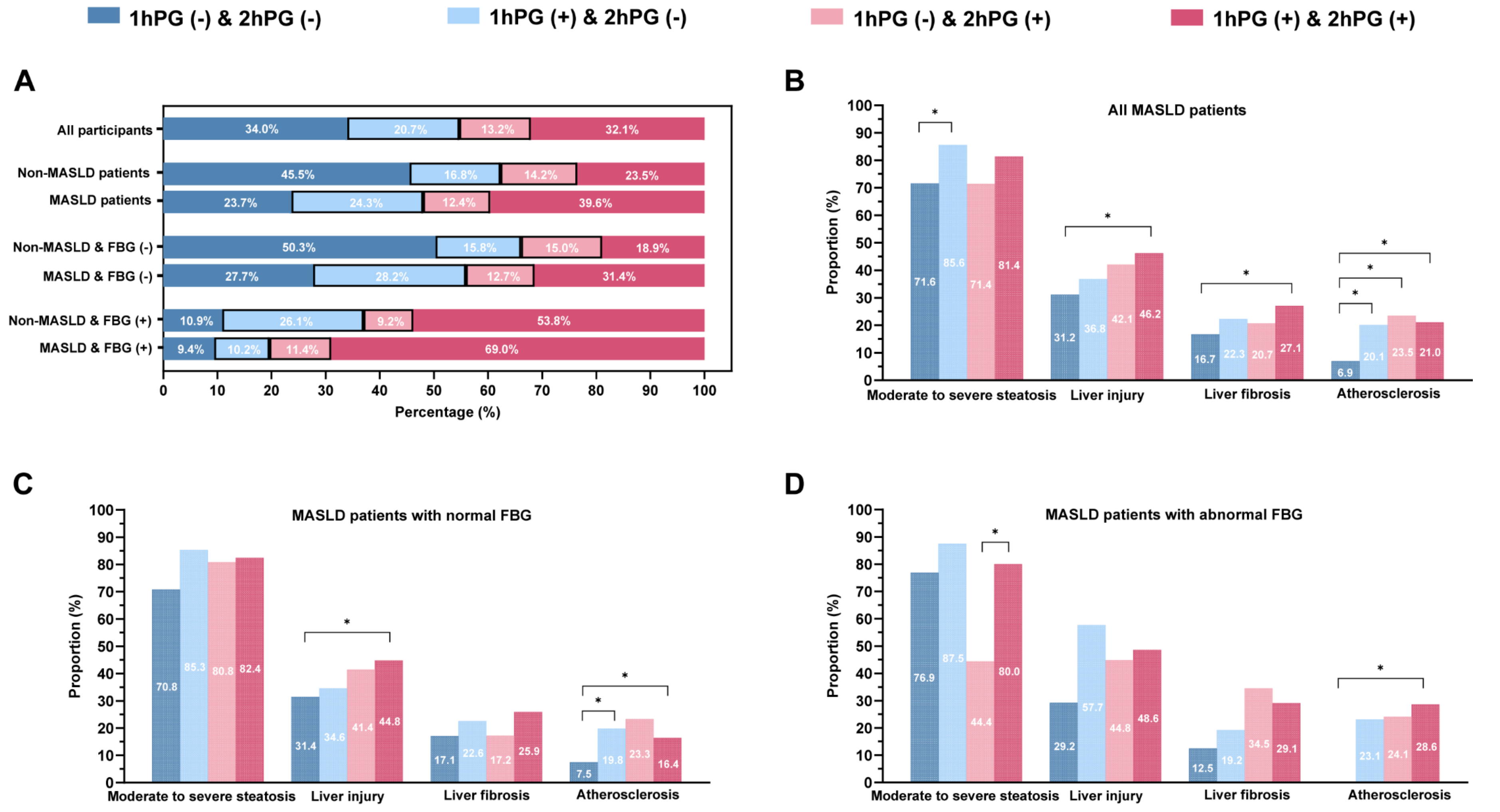

Figure S3. Prevalence of clinical outcomes in MASLD and NAFLD-non-MASLD subjects. OGTT cross group: 1hPG (-) & 2hPG (-) represents 1hPG and 2hPG are all normal. 1hPG (+) & 2hPG (-) indicates that 1hPG is abnormal, while 2hPG is normal. 1hPG (-) & 2hPG (+) indicates that 2hPG is abnormal, while 1hPG is normal. 1hPG (+) & 2hPG (+) represents 1hPG and 2hPG are all abnormal.

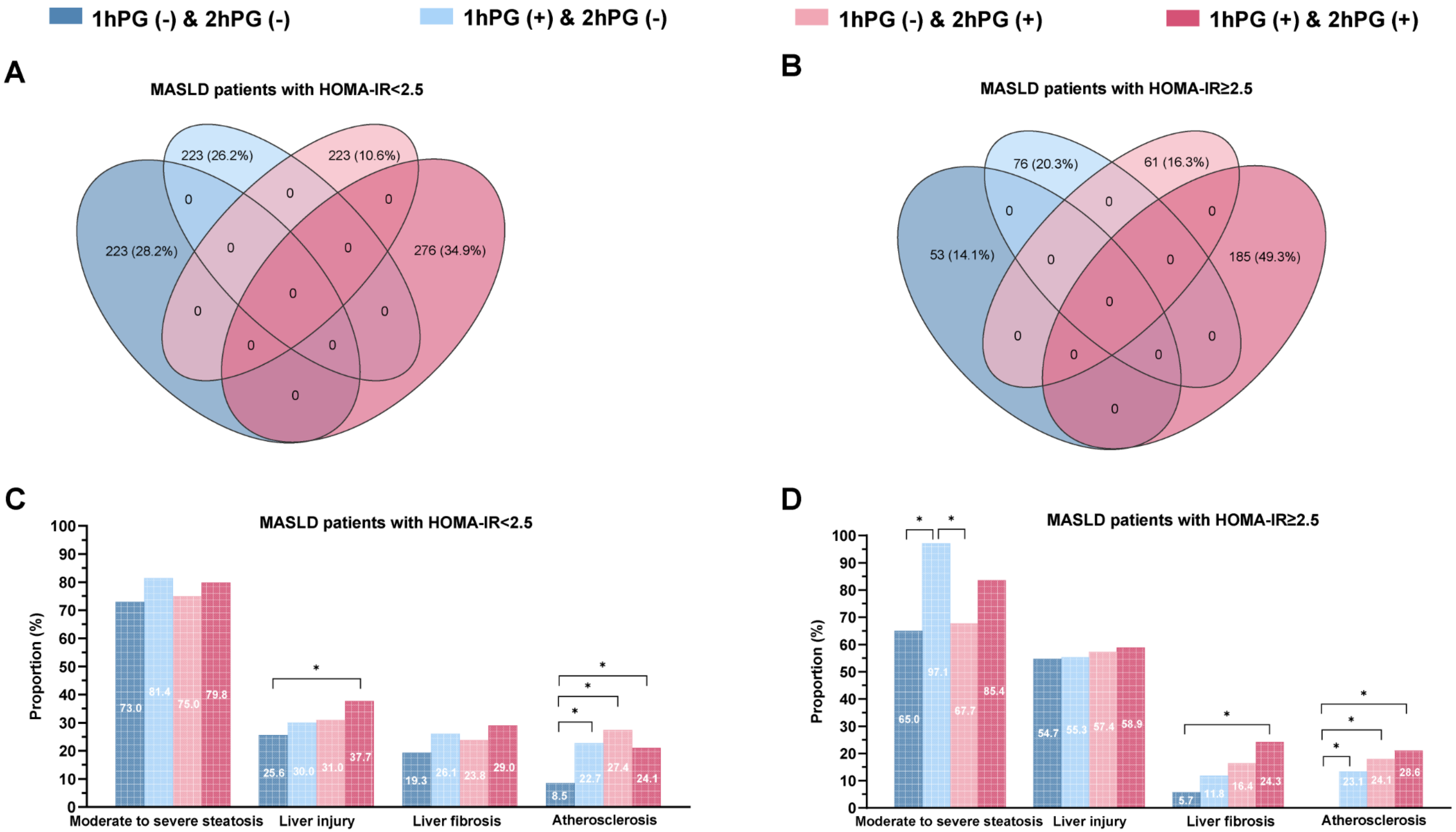

Figure S4. Prevalence of clinical outcomes in MASLD subjects among HOMA-IR subgroup. OGTT cross group: 1hPG (-) & 2hPG (-) represents 1hPG and 2hPG are all normal. 1hPG (+) & 2hPG (-) indicates that 1hPG is abnormal, while 2hPG is normal. 1hPG (-) & 2hPG (+) indicates that 2hPG is abnormal, while 1hPG is normal. 1hPG (+) & 2hPG (+) represents 1hPG and 2hPG are all abnormal.

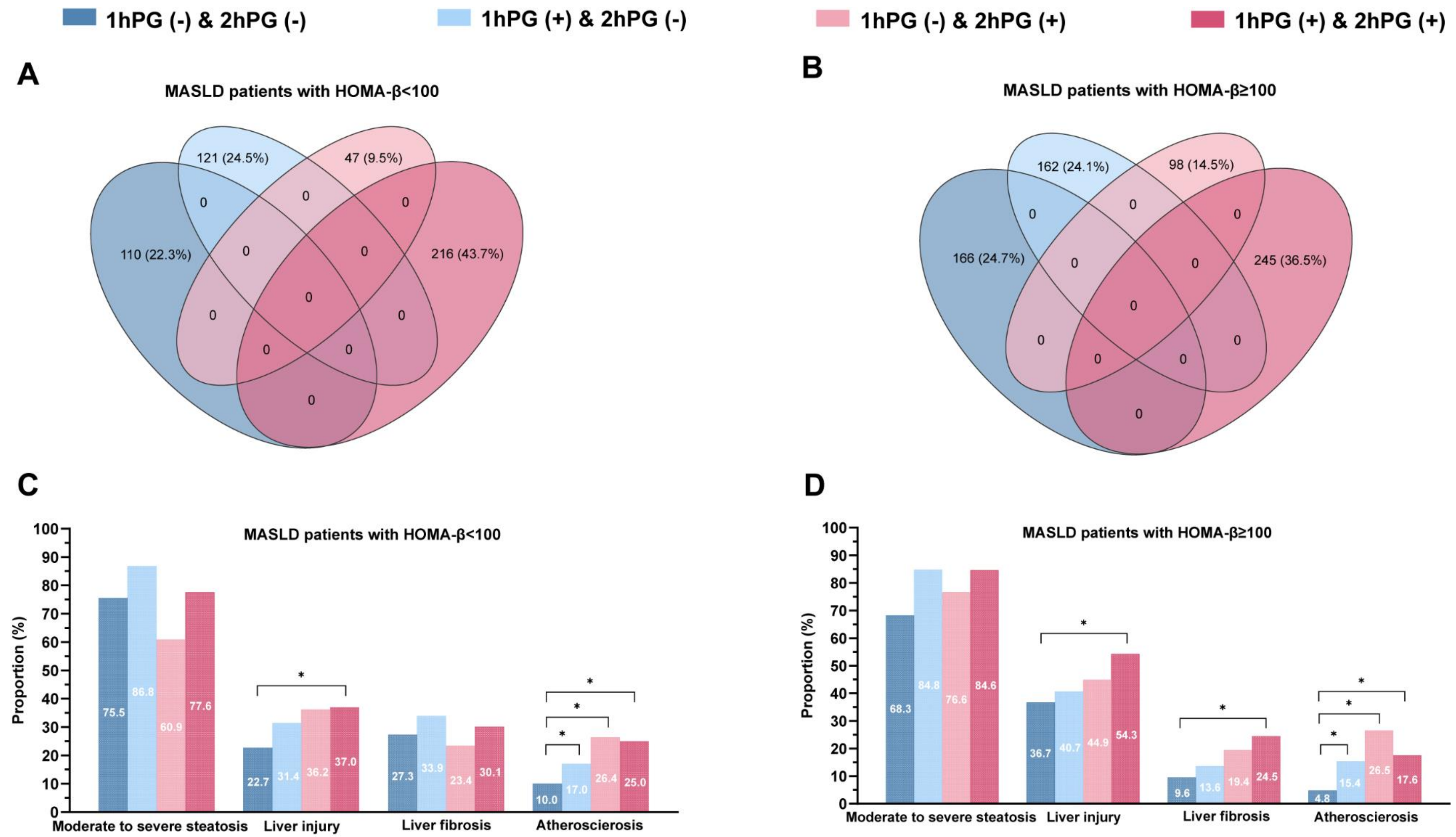

Figure S5. Prevalence of clinical outcomes in MASLD subjects among HOMA-β subgroup. OGTT cross group: 1hPG (-) & 2hPG (-) represents 1hPG and 2hPG are all normal. 1hPG (+) & 2hPG (-) indicates that 1hPG is abnormal, while 2hPG is normal. 1hPG (-) & 2hPG (+) indicates that 2hPG is abnormal, while 1hPG is normal. 1hPG (+) & 2hPG (+) represents 1hPG and 2hPG are all abnormal.

Table S1. Clinical characteristics of male MASLD patients.

Characteristics	1hPG (-) & 2hPG (-) (n = 116)	1hPG (+) & 2hPG (-) (n = 153)	1hPG (-) & 2hPG (+) (n = 91)	1hPG (+) & 2hPG (+) (n = 248)	*P*
Age (years)	38.90 ± 13.43	44.52 ± 14.40**a	45.62 ± 13.36**a	47.93 ± 12.21***a	<0.001
BMI (kg/m2)	26.82 ± 5.39	26.15 ± 5.95	27.77 ± 4.08**b	28.07 ± 4.59*a***b	0.001
WC (cm)	94.69 ± 15.11	92.37 ± 12.53	95.70 ± 10.72	96.59 ± 11.13**b	0.009
WHR	0.95 ± 0.19	0.93 ± 0.06	0.95 ± 0.06	0.96 ± 0.13	0.10
Hypertension, n (%)	62 (53.5%)	69 (45.1%)	63 (69.2%)*b	169 (68.2%)*a*b	<0.001
FBG (mmol/L)	4.60 (4.30,5.00)	4.90 (4.60,5.30)*a	4.80 (4.60,5.45)*a	5.30 (4.90,5.90)***a***b***c	<0.001
1hPG (mmol/L)	7.50 (6.85,8.00)	9.40 (9.00,11.00)***a	7.80 (7.50,8.20)***b	10.45 (9.40,13.00)***a**b***c	<0.001
2hPG (mmol/L)	6.50 (5.77,7.20)	6.80 (6.10,7.20)	9.60 (8.50,11.05) ***a***b	11.00 (9.20,12.93) ***a***b	<0.001
FINS (μU/mL)	7.60 (5.15,10.31)	8.44 (5.11,10.77)	8.98 (6.33,13.98)*a	8.98 (6.83,11.52)*a*b	0.002
HOMA-IR	1.68 (1.11,2.26)	1.81 (1.08,2.39)	2.04 (1.29,3.07)*a	2.11 (1.63,2.79) ***a***b	<0.001
HOMA-β (%)	95.91 (71.73,138.49)	114.25 (73.60,151.88)	138.27 (77.95,191.08)**a*b	131.57 (72.82,223.99) ***a***b	<0.001
HBA1c (%)	5.60 (5.30,5.80)	5.50 (5.20,5.73)	5.73 (5.50,6.05) *a***b	5.86 (5.60,6.30) ***a***b	<0.001
CHOL (mmol/L)	4.99 ± 1.20	4.84 ± 1.05	5.04 ± 1.20	5.01 ± 1.24	0.49
TG (mmol/L)	1.37 (1.02,1.93)	1.38 (1.06,2.05)	1.87 (1.25,2.72) **a**b	1.76 (1.30,2.54) ***a***b	<0.001
HDL-C (mmol/L)	1.15 ± 0.30	1.12 ± 0.27	1.02 ± 0.25**a*b	1.06 ± 0.23*a	<0.001
LDL-C (mmol/L)	3.20 ± 0.87	3.10 ± 0.85	3.22 ± 0.88	3.19 ± 0.90	0.71
Uric acid (μmol/L)	430.21 ± 114.31	427.88 ± 102.86	455.88 ± 132.65	447.57 ± 107.74	0.13
ALT (U/L)	27.00 (19.00,40.00)	30.00 (19.00,45.00)	28.00 (20.00,46.00)	32.00 (23.75,47.00)*a	0.02
AST (U/L)	22.50 (19.00,28.00)	24.00 (19.00,30.00)	23.00 (19.00,32.50)	25.00 (21.00,32.00)	0.30
ALP (U/L)	76.50 (63.25,90.00)	76.00 (65.00,89.00)	72.00 (63.00,86.00)	74.00 (63.00,90.00)	0.83
Albumin (g/L)	42.25 (39.98,44.92)	41.70 (39.60,44.00)	42.00 (38.80,43.90)	41.85 (39.80,44.40)	0.61
CT attenuation (HU)	32.25 (30.88,38.94)	32.50 (28.38,36.25)	33.12 (31.88,43.00)	32.50 (26.88,34.88)	0.23
Platelet (10^9/L)	251.87 ± 61.93	234.60 ± 56.25	265.97 ± 82.65 **a*b	246.76 ± 74.98	0.007
FIB-4 index	0.67 (0.45,1.00)	0.81 (0.57,1.15)	0.79 (0.51,1.15)	0.90 (0.62,1.34)***a	<0.001

Abbreviation: BMI, body mass index; WC, waist circumference; WHR, waist-to-hip ratio; FBG, fasting blood glucose; 1hPG, 1-hour post load plasma glucose; 2hPG, 2-hour post load plasma glucose; FINS, fasting insulin; HOMA-IR, homeostasis model assessment of insulin resistance; HOMA-β, homeostasis model assessment of β-cell function; HBA1c, glycosylated hemoglobin; CHOL, cholesterol; TG, triglyceride; HDL-C, high-density lipoprotein cholesterol; LDL-C, low-density lipoprotein cholesterol; ALT, alanine aminotransferase; AST, aspartate transaminase; ALP , alkaline phosphatase; FIB-4 index, fibrosis-4 index.

Table S2. Clinical characteristics of female MASLD patients.

Characteristics	1hPG (-) & 2hPG (-) (n = 160)	1hPG (+) & 2hPG (-) (n = 130)	1hPG (-) & 2hPG (+) (n = 54)	1hPG (+) & 2hPG (+) (n = 213)	*P*
Age (years)	43.06 ± 16.95	46.73 ± 15.97	46.80 ± 16.60	49.09 ± 13.69**a	0.003
BMI (kg/m2)	24.79 ± 5.43	25.56 ± 5.73	28.67 ± 4.52***a***b	27.65 ± 4.01***a***b	<0.001
WC (cm)	85.38 ± 11.63	88.08 ± 14.28	94.89 ± 10.78***a***b	92.14 ± 9.08***a***b	<0.001
WHR	0.88 ± 0.06	0.89 ± 0.07	0.91 ± 0.06**a	0.92 ± 0.06***a**b	<0.001
Hypertension, n (%)	56 (35.0%)	49 (37.7%)	30 (55.6%)*a	131 (61.5%)*a*b	<0.001
FBG (mmol/L)	4.60 (4.30,4.90)	4.90 (4.60,5.20)***a	4.90 (4.70,5.40)***a	5.30 (4.90,6.00) ***a***b*c	<0.001
1hPG (mmol/L)	7.50 (6.60,8.00)	9.30 (8.90,10.30)***a	7.90 (7.55,8.20)***b	10.50 (9.40,13.00) ***a***b***c	<0.001
2hPG (mmol/L)	6.30 (5.27,7.10)	6.85 (6.20,7.40)	9.45 (8.43,10.57) ***a***b	11.20 (9.00,13.60) ***a***b	<0.001
FINS (μU/mL)	7.24 (4.46,11.28)	8.71 (5.00,12.50)	10.28 (8.30,17.11) ***a**b	10.35 (7.79,14.47) ***a**b	<0.001
HOMA-IR	1.56 (0.89,2.28)	1.86 (1.05,2.75)	2.28 (1.64,3.75) ***a*b	2.49 (1.76,4.06) ***a***b	<0.001
HOMA-β (%)	119.58 (80.89,202.30)	109.72 (74.54,188.03)	137.07 (99.50,217.23)	117.00 (79.76,173.20)	0.37
HBA1c (%)	5.40 (5.10,5.73)	5.55 (5.30,5.80)	5.77 (5.42,6.09) ***a*b	5.83 (5.60,6.20) ***a***b	<0.001
CHOL (mmol/L)	5.05 ± 1.05	5.16 ± 1.12	5.27 ± 1.32	5.17 ± 1.19	0.62
TG (mmol/L)	1.19 (0.87,1.61)	1.19 (0.94,1.62)	1.84 (1.45,2.25) ***a***b	1.59 (1.21,2.25) ***a***b	<0.001
HDL-C (mmol/L)	1.34 ± 0.35	1.32 ± 0.33	1.13 ± 0.27***a**b	1.18 ± 0.29***a***b	<0.001
LDL-C (mmol/L)	3.13 ± 0.75	3.22 ± 0.86	3.30 ± 0.76	3.28 ± 0.86	0.28
Uric acid (μmol/L)	352.59 ± 104.83	360.03 ± 124.29	413.56 ± 95.46***a**b	387.19 ± 105.48**a*b	<0.001
ALT (U/L)	17.00 (13.00,27.00)	19.00 (15.00,29.00)	25.00 (17.00,35.50)**a	24.00 (16.00,35.00) ***a	<0.001
AST (U/L)	19.50 (16.00,23.00)	21.00 (18.00,27.00)	20.50 (17.00,25.75)	21.00 (17.00,28.00) **a	0.01
ALP (U/L)	70.00 (59.00,84.00)	68.00 (60.00,81.75)	79.50 (67.25,87.00)	74.00 (63.00,88.00)	0.20
Albumin (g/L)	40.15 (38.05,42.42)	41.00 (38.73,43.40)	41.20 (38.52,44.00)	40.80 (38.30,43.00)	0.22
Platelet (10^9/L)	259.84 ± 65.14	259.59 ± 64.08	278.91 ± 84.16	267.40 ± 75.73	0.28
CT attenuation (HU)	33.00 (31.12,42.25)	32.38 (30.56,34.31)	31.38 (24.56,36.06)	32.12 (22.06,34.06)	0.36
FIB-4 index	0.79 (0.48,1.09)	0.94 (0.54,1.27)	0.63 (0.48,1.00)	0.85 (0.60,1.19)	0.40

Abbreviation: BMI, body mass index; WC, waist circumference; WHR, waist-to-hip ratio; FBG, fasting blood glucose; 1hPG, 1-hour post load plasma glucose; 2hPG, 2-hour post load plasma glucose; FINS, fasting insulin; HOMA-IR, homeostasis model assessment of insulin resistance; HOMA-β, homeostasis model assessment of β-cell function; HBA1c, glycosylated hemoglobin; CHOL, cholesterol; TG, triglyceride; HDL-C, high-density lipoprotein cholesterol; LDL-C, low-density lipoprotein cholesterol; ALT, alanine aminotransferase; AST, aspartate transaminase; ALP , alkaline phosphatase; FIB-4 index, fibrosis-4 index.

Table S3. Clinical characteristics of MASLD aged ≥40 years old.

Characteristics	1hPG (-) & 2hPG (-) (n = 137)	1hPG (+) & 2hPG (-) (n = 179)	1hPG (-) & 2hPG (+) (n = 91)	1hPG (+) & 2hPG (+) (n = 334)	*P*
Age (years)	54.47 ± 10.16	55.12 ± 9.20	55.05 ± 9.84	54.55 ± 9.18	0.89
Male, n (%)	49 (35.8%)	96 (53.6%)*a	58 (63.7%)*a	179 (53.6%)*a	<0.001
BMI (kg/m2)	24.51 ± 3.75	24.75 ± 4.50	27.17 ± 3.20***a***b	27.40 ± 3.80***a***b	<0.001
WC (cm)	87.52 ± 12.66	88.86 ± 10.74	93.74 ± 8.21***a***b	94.09 ± 9.54***a***b	<0.001
WHR	0.92 ± 0.18	0.92 ± 0.06	0.93 ± 0.06**a	0.94 ± 0.12***a**b	0.03
Hypertension, n (%)	62 (45.3%)	83 (46.4%)	61 (67.0%)*a*b	233 (69.8%)*a*b	<0.001
FBG (mmol/L)	4.70 (4.40,5.00)	5.00 (4.65,5.30)**a	5.00 (4.70,5.60)***a	5.40 (5.00,6.00) ***a***b***c	<0.001
1hPG (mmol/L)	7.50 (6.70,8.10)	9.70 (9.00,11.35)***a	7.90 (7.45,8.20)***b	11.00 (9.40,13.20) ***a**b***c	<0.001
2hPG (mmol/L)	6.40 (5.60,7.20)	6.90 (6.10,7.30)	9.70 (8.60,11.30) ***a***b	11.20 (9.43,13.80) ***a***b	<0.001
FINS (μU/mL)	5.60 (4.37,8.57)	7.24 (4.58,10.16)*a	8.61 (6.11,11.79) ***a*b	8.98 (6.93,12.09) ***a***b	<0.001
HOMA-IR	1.20 (0.84,1.74)	1.62 (1.00,2.32)**a	1.92 (1.34,2.68) ***a*b	2.19 (1.67,3.07) ***a***b	<0.001
HOMA-β (%)	89.83 (66.77,146.86)	95.83 (66.20,138.78)	111.00 (74.87,165.30)	95.91 (63.93,137.91)	0.27
HBA1c (%)	5.60 (5.30,5.80)	5.50 (5.30,5.80)	5.80 (5.60,6.15)***a***b	5.90 (5.60,6.40) ***a***b	<0.001
CHOL (mmol/L)	5.09 ± 1.11	4.99 ± 1.15	5.02 ± 1.27	5.05 ± 1.22	0.88
TG (mmol/L)	1.19 (0.94,1.59)	1.28 (0.96,1.62)	1.92 (1.32,2.71) ***a***b	1.67 (1.25,2.35) ***a***b	<0.001
HDL-C (mmol/L)	1.32 ± 0.36	1.23 ± 0.30	1.04 ± 0.22***a***b	1.13 ± 0.27***a***b	<0.001
LDL-C (mmol/L)	3.15 ± 0.79	3.17 ± 0.90	3.18 ± 0.82	3.21 ± 0.88	0.92
Uric acid (μmol/L)	357.19 ± 103.09	377.87 ± 118.33	423.79 ± 125.68***a*b	406.06 ± 104.15***a*b	<0.001
ALT (U/L)	20.00 (15.00,28.00)	22.00 (17.00,32.00)	22.00 (17.00,33.00)	27.00 (19.00,36.00) ***a*b	<0.001
AST (U/L)	21.00 (17.00,24.00)	22.00 (18.50,27.50)	21.00 (17.00,26.00)	23.00 (19.00,29.00)**a	0.001
ALP (U/L)	69.00 (58.00,82.00)	73.00 (61.50,82.00)	70.00 (64.00,86.00)	74.00 (64.00,90.00)	0.11
Albumin (g/L)	40.00 (37.70,42.40)	40.66 (38.60,43.00)	40.80 (37.70,42.65)	41.00 (38.73,43.65)	0.20
Platelet (10^9/L)	247.93 ± 60.71	233.96 ± 60.50	264.19 ± 89.98 *a***b	242.95 ± 65.52	0.005
CT attenuation (HU)	32.50 (31.12,41.25)	32.50 (27.50,35.06)	32.75 (30.25,42.25)	32.00 (23.00,33.75)	0.12
FIB-4 index	0.98 (0.79,1.42)	1.08 (0.85,1.41)	0.92 (0.75,1.37)	1.07 (0.78,1.44)	0.11

Abbreviation: BMI, body mass index; WC, waist circumference; WHR, waist-to-hip ratio; FBG, fasting blood glucose; 1hPG, 1-hour post load plasma glucose; 2hPG, 2-hour post load plasma glucose; FINS, fasting insulin; HOMA-IR, homeostasis model assessment of insulin resistance; HOMA-β, homeostasis model assessment of β-cell function; HBA1c, glycosylated hemoglobin; CHOL, cholesterol; TG, triglyceride; HDL-C, high-density lipoprotein cholesterol; LDL-C, low-density lipoprotein cholesterol; ALT, alanine aminotransferase; AST, aspartate transaminase; ALP , alkaline phosphatase; FIB-4 index, fibrosis-4 index.

Table S4. Clinical characteristics of MASLD aged < 40 years old.

Characteristics	1hPG (-) & 2hPG (-) (n = 139)	1hPG (+) & 2hPG (-) (n = 104)	1hPG (-) & 2hPG (+) (n = 54)	1hPG (+) & 2hPG (+) (n = 127)	*P*
Age (years)	28.34 ± 6.84	29.04 ± 6.91	30.89 ± 6.48	32.47 ± 5.51***a**b	<0.001
Male, n (%)	67 (48.2%)	57 (54.8%)	33 (61.1%)	69 (54.3%)	0.40
BMI (kg/m2)	26.76 ± 6.63	27.81 ± 7.26	29.67 ± 5.28**a*b	29.13 ± 5.31**a	0.004
WC (cm)	91.04 ± 14.97	93.05 ± 17.00	98.18 ± 13.59**a*b	95.71 ± 12.54	0.007
WHR	0.90 ± 0.07	0.90 ± 0.08	0.94 ± 0.07*a*b	0.93 ± 0.06**a**b	<0.001
Hypertension, n (%)	56 (40.3%)	35 (33.7%)	32 (59.3%)*b	67 (52.8%)*b	0.003
FBG (mmol/L)	4.60 (4.30,4.80)	4.80 (4.47,5.23)**a	4.80 (4.60,5.00)	5.00 (4.70,5.45)***a**b*c	<0.001
1hPG (mmol/L)	7.50 (6.60,7.95)	9.20 (8.80,9.70)***a	7.80 (7.60,8.20)***b	9.80 (9.50,11.90)***a**b***c	<0.001
2hPG (mmol/L)	6.40 (5.30,7.10)	6.60 (6.10,7.10)	9.15 (8.43,10.10) ***a***b	10.20 (8.70,11.90)***a***b	<0.001
FINS (μU/mL)	9.01 (5.28,13.65)	9.50 (6.27,14.26)	13.18 (9.00,18.20)**a*b	10.14 (7.63,15.72)*a	0.001
HOMA-IR	1.96 (1.18,2.72)	2.12 (1.26,3.13)	2.79 (1.90,4.18)**a	2.35 (1.72,4.21)**a	<0.001
HOMA-β (%)	140.25 (91.24,224.57)	142.25 (108.33,241.29)	183.89 (137.07,284.28)	174.88 (103.90,273.40)	0.052
HBA1c (%)	5.40 (5.10,5.73)	5.55 (5.24,5.73)	5.71 (5.40,5.86)**a	5.73 (5.49,6.00)***a**b	<0.001
CHOL (mmol/L)	4.96 ± 1.12	4.99 ± 0.99	5.31 ± 1.20	5.17 ± 1.20	0.14
TG (mmol/L)	1.31 (0.95,1.76)	1.37 (1.06,2.37)	1.78 (1.31,2.25)**a	1.60 (1.21,2.58)***a	<0.001
HDL-C (mmol/L)	1.20 ± 0.31	1.18 ± 0.34	1.08 ± 0.31*a	1.09 ± 0.27*a	0.01
LDL-C (mmol/L)	3.16 ± 0.81	3.14 ± 0.78	3.35 ± 0.86	3.31 ± 0.87	0.21
Uric acid (μmol/L)	412.83 ± 120.27	429.14 ± 110.57	467.62 ± 109.95*a	455.48 ± 119.75	0.004
ALT (U/L)	21.00 (14.00,37.50)	30.00 (16.75,48.75)	35.00 (22.25,59.50)**a	34.00 (23.50,56.00)***a	<0.001
AST (U/L)	21.00 (17.00,26.50)	24.50 (17.00,32.00)	25.00 (20.00,36.00)*a	25.00 (19.00,36.00)**a	0.002
ALP (U/L)	76.00 (64.00,90.50)	72.00 (63.75,90.00)	80.50 (70.00,91.50)	78.00 (62.50,89.00)	0.58
Albumin (g/L)	42.00 (39.80,44.80)	43.05 (40.80,44.65)	42.85 (41.00,44.92)	42.10 (40.35,44.60)	0.33
Platelet (10^9/L)	264.93 ± 65.87	266.94 ± 56.71	281.92 ± 69.57***a***b	291.39 ± 89.45***a***b	0.01
CT attenuation (HU)	33.00 (31.06,41.44)	32.50 (30.19,36.19)	32.50 (30.00,42.25)	32.75 (27.56,38.00)	0.86
FIB-4 index	0.47 (0.37,0.71)	0.49 (0.36,0.65)	0.48 (0.39,0.61)	0.48 (0.40,0.64)	0.94

Abbreviation: BMI, body mass index; WC, waist circumference; WHR, waist-to-hip ratio; FBG, fasting blood glucose; 1hPG, 1-hour post load plasma glucose; 2hPG, 2-hour post load plasma glucose; FINS, fasting insulin; HOMA-IR, homeostasis model assessment of insulin resistance; HOMA-β, homeostasis model assessment of β-cell function; HBA1c, glycosylated hemoglobin; CHOL, cholesterol; TG, triglyceride; HDL-C, high-density lipoprotein cholesterol; LDL-C, low-density lipoprotein cholesterol; ALT, alanine aminotransferase; AST, aspartate transaminase; ALP , alkaline phosphatase; FIB-4 index, fibrosis-4 index.

Table S5. Clinical characteristics of MASLD with FBG ≥5.6 mmol/L.

Characteristics	1hPG (-) & 2hPG (-) (n = 24)	1hPG (+) & 2hPG (-) (n = 26)	1hPG (-) & 2hPG (+) (n = 29)	1hPG (+) & 2hPG (+) (n = 175)	*P*
Age (years)	43.17 ± 13.81	43.38 ± 17.23	52.45 ± 14.21	50.02 ± 12.38	0.07
Male, n (%)	15 (62.5%)	17 (65.4%)	20 (69.0%)	92 (52.6%)	0.25
BMI (kg/m2)	25.56 ± 4.64	28.34 ± 5.53	27.66 ± 3.69	28.30 ± 4.31	0.08
WC (cm)	87.42 ± 13.33	96.90 ± 12.97*a	96.53 ± 10.30**a	95.54 ± 11.23**a	0.008
WHR	0.92 ± 0.05	0.92 ± 0.06	0.95 ± 0.06	0.94 ± 0.07	0.20
Hypertension, n (%)	14 (58.3%)	13 (50.0%)	21 (72.4%)	117 (66.9%)	0.26
FBG (mmol/L)	5.90 (5.80,6.03)	5.80 (5.70,5.97)	5.80 (5.70,6.40)	6.00 (5.80,6.70)**b	0.001
1hPG (mmol/L)	7.90 (7.35,8.30)	9.25 (9.12,10.75)**a	7.90 (7.70,8.10)**b	12.70 (10.00,14.95)***a***b***c	<0.001
2hPG (mmol/L)	7.10 (6.50,7.30)	6.60 (5.98,7.00)	10.30 (9.40,13.20)***a***b	13.30 (10.15,15.10)***a***b	<0.001
FINS (μU/mL)	7.00 (5.35,8.79)	9.75 (7.69,13.13)*a	10.22 (8.33,18.11)*a	9.18 (7.33,14.07)*a	0.01
HOMA-IR	1.82 (1.42,2.45)	2.51 (1.96,3.31)	2.73 (2.11,4.83)*a	2.67 (2.09,4.28)**a	0.005
HOMA-β (%)	59.98 (43.25,71.35)	79.71 (67.46,123.66)*a	81.76 (47.52,144.88)**a	72.00 (52.46,99.47)	0.02
HBA1c (%)	5.87 (5.29,6.50)	5.71 (5.60,5.86)	6.10 (5.80,7.00)**b	6.27 (5.86,6.89)***b	<0.001
CHOL (mmol/L)	5.01 ± 1.32	5.22 ± 1.22	5.05 ± 1.44	5.08 ± 1.33	0.95
TG (mmol/L)	1.37 (1.10,1.58)	1.36 (1.08,2.02)	1.93 (1.47,3.23)*a	1.80 (1.31,2.55)*a	0.002
HDL-C (mmol/L)	1.23 ± 0.27	1.20 ± 0.27	1.06 ± 0.30	1.09 ± 0.23	0.20
LDL-C (mmol/L)	3.09 ± 0.81	3.37 ± 0.90	3.18 ± 0.89	3.25 ± 0.92	0.74
Uric acid (μmol/L)	385.29 ± 149.63	409.69 ± 123.13	407.52 ± 112.44	418.86 ± 121.89	0.65
ALT (U/L)	24.00 (17.50,30.00)	41.00 (19.25,69.75)	27.00 (20.00,40.00)	30.00 (20.00,43.50)	0.18
AST (U/L)	21.50 (19.00,25.25)	29.00 (18.75,36.75)	23.00 (20.00,35.00)	24.00 (19.00,34.00)	0.38
ALP (U/L)	72.00 (61.75,81.00)	78.50 (70.00,95.75)	70.00 (64.00,86.00)	77.00 (65.50,90.00)	0.21
Albumin (g/L)	40.00 (37.75,42.28)	41.85 (39.20,45.20)	42.20 (39.60,43.60)	41.70 (40.05,44.50)	0.18
CT attenuation (HU)	32.25 (32.00,38.75)	32.75 (30.75,33.75)	42.50 (32.56,43.38)	32.00 (22.00,33.50)**c	0.01
Platelet (10^9/L)	257.88 ± 75.83	241.46 ± 62.24	276.14 ± 135.87	256.34 ± 73.23	0.47
FIB-4 index	0.85 (0.66,1.05)	0.79 (0.41,1.19)	0.92 (0.66,1.48)	0.93 (0.66,1.37)	0.44

Abbreviation: BMI, body mass index; WC, waist circumference; WHR, waist-to-hip ratio; FBG, fasting blood glucose; 1hPG, 1-hour post load plasma glucose; 2hPG, 2-hour post load plasma glucose; FINS, fasting insulin; HOMA-IR, homeostasis model assessment of insulin resistance; HOMA-β, homeostasis model assessment of β-cell function; HBA1c, glycosylated hemoglobin; CHOL, cholesterol; TG, triglyceride; HDL-C, high-density lipoprotein cholesterol; LDL-C, low-density lipoprotein cholesterol; ALT, alanine aminotransferase; AST, aspartate transaminase; ALP , alkaline phosphatase; FIB-4 index, fibrosis-4 index.

Table S6. Clinical characteristics of MASLD with FBG < 5.6 mmol/L.

**Characteristics**	**1hPG (-) & 2hPG (-) (n = 252)**	**1hPG (+) & 2hPG (-) (n = 257)**	**1hPG (-) & 2hPG (+) (n = 116)**	**1hPG (+) & 2hPG (+) (n = 286)**	***P***
Age (years)	41.13 ± 15.86	45.75 ± 14.95**a	44.46 ± 14.32	47.52 ± 13.16***a	<0.001
Male, n (%)	101 (40.1%)	136 (52.9%)*a	71 (61.2%)*a	156 (54.6%)*a	<0.001
BMI (kg/m2)	25.65 ± 5.58	25.63 ± 5.83	28.21 ± 4.40***a***b	27.62 ± 4.34***a***b	<0.001
WC (cm)	89.47 ± 14.03	89.74 ± 13.41	95.12 ± 10.84***a***b	93.92 ± 9.93***a***b	<0.001
WHR	0.91 ± 0.14	0.91 ± 0.07	0.93 ± 0.06**a	0.94 ± 0.13***a***b	<0.001
Hypertension, n (%)	104 (41.3%)	105 (40.9%)	72 (62.1%)*a*b	183 (64.0%)*a*b	<0.001
FBG (mmol/L)	4.60 (4.30,4.80)	4.90 (4.50,5.20)***a	4.80 (4.50,5.00)***a	5.00 (4.70,5.20) ***a**c	<0.001
1hPG (mmol/L)	7.50 (6.60,8.00)	9.40 (8.90,10.60)***a	7.80 (7.50,8.20)***b	9.80 (9.22,11.40) ***a***c	<0.001
2hPG (mmol/L)	6.30 (5.40,7.00)	6.80 (6.10,7.20)*a	9.20 (8.40,10.43) ***a***b	10.20 (8.70,12.00) ***a***b	<0.001
FINS (μU/mL)	7.47 (4.57,11.30)	8.05 (4.94,11.60)	9.40 (6.91,15.01) ***a**b	8.98 (6.87,12.51) ***a***b	<0.001
HOMA-IR	1.58 (0.92,2.26)	1.74 (1.01,2.50)	2.02 (1.43,2.93) ***a**b	2.01 (1.49,2.77) ***a***b	<0.001
HOMA-β (%)	128.21 (94.53,181.96)	115.00 (76.75,169.64)	144.36 (101.06,233.23)*a*b	133.17 (86.98,226.07)*a*b	0.002
HBA1c (%)	5.50 (5.20,5.73)	5.50 (5.22,5.73)	5.73 (5.40,5.86) ***a***b	5.73 (5.49,5.99) ***a***b	<0.001
CHOL (mmol/L)	5.03 ± 1.10	4.97 ± 1.08	5.15 ± 1.20	5.09 ± 1.14	0.44
TG (mmol/L)	1.27 (0.94,1.70)	1.30 (0.98,1.84)	1.81 (1.27,2.46) ***a***b	1.60 (1.20,2.36) ***a***b	<0.001
HDL-C (mmol/L)	1.26 ± 0.35	1.21 ± 0.32	1.06 ± 0.25***a***b	1.13 ± 0.28***a*b	<0.001
LDL-C (mmol/L)	3.16 ± 0.80	3.14 ± 0.85	3.26 ± 0.82	3.23 ± 0.86	0.43
Uric acid (μmol/L)	385.21 ± 111.85	395.40 ± 117.60	448.26 ± 122.83***a***b	420.18 ± 103.59**a	<0.001
ALT (U/L)	20.00 (14.00,32.00)	23.00 (17.00,36.00)	26.50 (17.75,42.25)**a	28.00 (20.00,40.00) ***a*b	<0.001
AST (U/L)	21.00 (17.00,25.00)	22.00 (18.00,28.00)	22.00 (17.00,30.00)	23.00 (19.00,29.00)**a	0.002
ALP (U/L)	74.00 (60.00,88.00)	72.00 (61.00,85.00)	75.00 (66.75,87.50)	73.00 (62.00,88.75)	0.29
Albumin (g/L)	41.15 (38.77,43.62)	41.50 (39.10,43.60)	41.45 (38.68,44.20)	41.00 (38.40,43.68)	0.67
CT attenuation (HU)	33.00 (30.94,41.50)	32.38 (27.81,35.25)	32.12 (25.88,34.19)	32.38 (26.81,35.44)	0.25
Platelet (10^9/L)	256.36 ± 62.73	246.55 ± 61.14	269.45 ± 64.47*a*b	256.27 ± 77.69	0.02
FIB-4 index	0.72 (0.45,1.09)	0.86 (0.56,1.23)*a	0.75 (0.49,1.00)	0.82 (0.59,1.30)**a	<0.001

Abbreviation: BMI, body mass index; WC, waist circumference; WHR, waist-to-hip ratio; FBG, fasting blood glucose; 1hPG, 1-hour post load plasma glucose; 2hPG, 2-hour post load plasma glucose; FINS, fasting insulin; HOMA-IR, homeostasis model assessment of insulin resistance; HOMA-β, homeostasis model assessment of β-cell function; HBA1c, glycosylated hemoglobin; CHOL, cholesterol; TG, triglyceride; HDL-C, high-density lipoprotein cholesterol; LDL-C, low-density lipoprotein cholesterol; ALT, alanine aminotransferase; AST, aspartate transaminase; ALP , alkaline phosphatase; FIB-4 index, fibrosis-4 index.

Table S7. Subgroup logistic regression analysis of the relationships between glucose status and clinical outcomes stratified by gender [a].

**Characteristics**	**1hPG (-) & 2hPG (-) (n = 116)**	**1hPG (+) & 2hPG (-) (n = 153)**	**1hPG (-) & 2hPG (+) (n = 91)**	**1hPG (+) & 2hPG (+) (n = 248)**	***P* for trend**	***P* for interaction**
Liver steatosis						0.33
Male	Reference	1.46 (0.56 - 3.79)	0.49 (0.18 - 1.31)	0.94 (0.37 - 2.37)	0.47	
Female	Reference	**2.95 (1.10 - 7.89)**	1.29 (0.39 - 4.30)	1.88 (0.78 - 4.49)	0.27	
Liver injury						0.26
Male	Reference	**1.71 (1.01 - 2.91)**	1.44 (0.78 - 2.67)	**2.17 (1.31 - 3.62)**	0.006	
Female	Reference	1.17 (0.69 - 1.95)	**2.01 (1.03 - 3.94)**	**2.05 (1.28 - 3.29)**	0.001	
Liver fibrosis						0.23
Male	Reference	1.37 (0.70 - 2.67)	**2.42 (1.14 - 5.14)**	**3.64 (1.96 - 6.77)**	<0.001	
Female	Reference	1.67 (0.93 - 2.99)	1.61 (0.68 - 3.79)	**1.93 (1.09 - 3.41)**	0.04	
Atherosclerosis						0.36
Male	Reference	1.82 (0.86 - 3.87)	**2.94 (1.32 - 6.56)**	1.94 (0.95 - 3.98)	0.15	
Female	Reference	**5.44 (2.15 - 13.75)**	2.44 (0.68 - 8.80)	**3.69 (1.46 - 9.33)**	0.06	

[a] adjusted for age, BMI, triglycerides, HDL-C, uric acid, and ALT (ALT except for liver injury; Age and ALT except for liver fibrosis).

Highlights refers odds ratios with significant differences ($p<0.05$).

Table S8. Subgroup logistic regression analysis of the relationships between glucose status and clinical outcomes stratified by age [a].

Characteristics	1hPG (-) & 2hPG (-) (n = 160)	1hPG (+) & 2hPG (-) (n = 130)	1hPG (-) & 2hPG (+) (n = 54)	1hPG (+) & 2hPG (+) (n = 213)	***P* for trend**	***P* for interaction**
Liver steatosis						0.45
Age ≥ 40 years	Reference	1.99 (0.81 - 4.84)	0.63 (0.22 - 1.78)	1.24 (0.52 - 2.96)	0.94	
Age < 40 years	Reference	2.13 (0.76 - 6.00)	0.81 (0.29 - 2.26)	1.46 (0.58 - 3.67)	0.74	
Liver injury						0.38
Age ≥ 40 years	Reference	1.13 (0.68 - 1.87)	0.94 (0.50 - 1.75)	**1.70 (1.07 - 2.71)**	0.008	
Age < 40 years	Reference	1.66 (0.97 - 2.85)	**2.72 (1.37 - 5.38)**	**2.28 (1.36 - 3.83)**	0.001	
Liver fibrosis						0.04
Age ≥ 40 years	Reference	1.18 (0.72 - 1.93)	1.45 (0.78 - 2.69)	**1.72 (1.07 - 2.75)**	0.02	
Age < 40 years	Reference	0.61 (0.05 - 7.00)	2.78 (0.33 - 23.19)	4.12 (0.75 - 22.64)	0.050	
Atherosclerosis						0.23
Age ≥ 40 years	Reference	**2.34 (1.28 - 4.28)**	**2.26 (1.10 - 4.63)**	**2.35 (1.31 - 4.21)**	0.03	
Age < 40 years	Reference	**10.51 (1.28 - 86.30)**	**24.79 (3.04 - 202.28)**	**8.63 (1.06 - 70.38)**	0.046	

[a] adjusted for sex, BMI, triglycerides, HDL-C, uric acid, and ALT (ALT except for liver injury; ALT except for liver fibrosis).

Highlights refers odds ratios with significant differences ($p<0.05$).

Table S9. Subgroup logistic regression analysis of the relationships between glucose status and clinical outcomes stratified by FBG [a].

**Characteristics**	**1hPG (-) & 2hPG (-) (n = 137)**	**1hPG (+) & 2hPG (-) (n = 179)**	**1hPG (-) & 2hPG (+) (n = 91)**	**1hPG (+) & 2hPG (+) (n = 334)**	***P* for trend**	***P* for interaction**
Liver steatosis						0.53
FBG ≥ 5.6 mmol/L	Reference	1.36 (0.16 - 11.48)	0.10 (0.01 - 0.66)	0.55 (0.11 - 2.83)	0.54	
FBG < 5.6mmol/L	Reference	**2.26 (1.12 - 4.57)**	1.51 (0.63 - 3.59)	1.59 (0.78 - 3.24)	0.36	
Liver injury						0.30
FBG ≥ 5.6 mmol/L	Reference	3.32 (0.95 - 11.66)	2.47 (0.71 - 8.57)	2.47 (0.90 - 6.82)	0.27	
FBG < 5.6mmol/L	Reference	1.26 (0.86 - 1.86)	1.42 (0.87 - 2.31)	**1.84 (1.25 - 2.70)**	0.002	
Liver fibrosis						0.13
FBG ≥ 5.6 mmol/L	Reference	2.12 (0.43 - 10.43)	**5.59 (1.24 - 25.11)**	**4.64 (1.24 - 17.32)**	0.02	
FBG < 5.6mmol/L	Reference	1.44 (0.91 - 2.27)	1.54 (0.82 - 2.88)	**2.34 (1.48 - 3.72)**	<0.001	
Atherosclerosis						0.39
FBG ≥ 5.6 mmol/L	Reference	**3.22 (1.65 - 6.26)**	1.71 (0.73 - 4.02)	2.52 (0.98 - 4.95)	0.11	
FBG < 5.6mmol/L	Reference	**2.37 (1.30 - 4.33)**	**3.29 (1.60 - 6.76)**	1.71 (0.91 - 3.19)	0.32	

[a] adjusted for sex, age, BMI, triglycerides, HDL-C, uric acid, and ALT (ALT except for liver injury; Age and ALT except for liver fibrosis).

Highlights refers odds ratios with significant differences ($p<0.05$).
